# Consistent expression of guanylyl cyclase-C in primary and metastatic gastrointestinal cancers

**DOI:** 10.1371/journal.pone.0189953

**Published:** 2017-12-19

**Authors:** Hadi Danaee, Thea Kalebic, Timothy Wyant, Matteo Fassan, Claudia Mescoli, Feng Gao, William L. Trepicchio, Massimo Rugge

**Affiliations:** 1 Millennium Pharmaceuticals, Inc., a wholly owned subsidiary of Takeda Pharmaceutical Company Limited, Cambridge, Massachusetts, United States of America; 2 Department of Medicine, DIMED, Surgical Pathology and Cytopathology Unit, University of Padua, Padua, Italy; National Cancer Center, JAPAN

## Abstract

**Background:**

The transmembrane receptor guanylate cyclase-C (GCC) has been found to be expressed in colorectal cancers. However, limited data are available on GCC protein expression in non-colorectal gastrointestinal tumors and few studies have reported whether GCC protein expression was consistently preserved in synchronous primary and metastatic cancer tissues.

**Methods:**

GCC protein status was assessed by immunohistochemistry in tumor specimens from individuals (n = 627) with gastrointestinal tumors, including esophageal (n = 130), gastric (n = 276), pancreatic (n = 136), and colorectal (n = 85) primary and metastatic tumors. Tissue specimens consisted of tissue microarrays containing esophageal, gastric, pancreatic tumors, and whole-slide tissue sections from colorectal cancer patients with matching primary and metastatic tumors

**Result:**

Among the evaluated esophageal, gastric, and pancreatic tumors, the frequency of GCC positivity at the protein level ranged from 59% to 68%. GCC was consistently expressed in primary and matched/synchronous metastatic lesions of colorectal cancer tissues derived from the same patients.

**Conclusion:**

This observational study demonstrated the protein expression of GCC across various gastrointestinal malignancies. In all cancer histotypes, GCC protein localization was observed predominantly in the cytoplasm compared to the membrane region of tumor cells. Consistent immunohistochemistry detection of GCC protein expression in primary colorectal cancers and in their matched liver metastases suggests that the expression of GCC is maintained throughout the process of tumor progression and formation of metastatic disease.

## Introduction

Guanylyl cyclase-C (GCC) is a transmembrane receptor protein encoded by the *GUCY2C* gene and activated by the endogenous hormones guanylin and uroguanylin, as well as bacterial heat-stable enterotoxins [1, 2]. GCC plays a central role in the regulation of intestinal electrolyte homeostasis [3]. It is also involved in mucosal barrier function, inflammation, and modulation of intestinal cell proliferation [4, 5].

In healthy individuals, GCC protein expression, which has been observed throughout the gut (as assessed by immunohistochemistry [IHC]) is restricted to the luminal side of the intestinal epithelium (from the duodenum to the rectum). This compartmentalization of GCC expression is preserved by the integrity of tight junctions [6]. GCC expression has also been detected in various gastrointestinal (GI) epithelial malignancies [7], including colorectal [8–10] and pancreatic [11, 12] adenocarcinomas. Recently, GCC has been exploited as a target for novel therapeutic modalities such as antibody-drug conjugates [13, 14]. However, data comparing GCC protein expression in primary and metastatic GI cancers remain limited [15] and observations from larger populations are sparse.

This study evaluated the protein expression of GCC in a series of tumors from 627 patients, among which 542 were individuals with non-colorectal malignancies, including esophageal, gastric, and pancreatic carcinomas. Additionally, we examined the GCC protein status of primary and metastatic tumors in a series of 85 matched primary colorectal cancers (CRC) and synchronous liver metastases.

## Materials and methods

### Tumor specimens

Formalin-fixed paraffin-embedded tissue microarrays for esophageal, gastric, and pancreatic tumors were procured from commercial sources (US Biomax, Rockville, MD, USA [arrays: STC1021, STC1501, ST8013, ST810a, PA1002, PA1921, and ES8010] and Pantomics, Richmond, CA, USA [arrays: PAC481 and ESC1021]). A total of 542 patient specimens (tumor cores) were examined. The study also assessed a series of primary CRC and matched synchronous liver-metastatic tissue specimens from 85 individuals (archival tissue specimens from the Surgical Pathology & Cytopathology Unit, Padua University; Ethics Committee #37593; 30/06/2011). All tissue specimens were anonymized prior to use in this study, and tumor subtypes were confirmed upon examination by two independent pathologists.

### GCC IHC analysis and pathology assessment

GCC staining was performed on the Techmate Automated IHC Platform (BioTek Solutions/Ventana Medical Systems, Tucson, AZ, USA; QualTek Laboratories, Goleta, CA, USA). In brief, 4 μm sections of tissue samples were dewaxed through xylene and a series of alcohol washes and finally placed into water. Antigen retrieval was performed using a steam heat-induced epitope retrieval (SHIER2) system (Black and Decker Steamer, Black & Decker, Baltimore, MD, USA). The rabbit monoclonal anti-GCC antibody used in this study (Millennium Pharmaceuticals, Inc., a wholly owned subsidiary of Takeda Pharmaceutical Company Limited, Cambridge, MA, USA; clone 148–2) was incubated overnight at 1 μg/mL, followed by incubation with a non-biotin peroxidase detection system (UltraVision, Thermo/Lab Vision, Freemont, CA, USA). Positive staining was detected using diaminobenzidine, and sections were counterstained with hematoxylin-eosin stain. Appropriate positive and negative control samples consisting of both colonic tissues, as well as GCC-transfected cell lines, were run concurrently (data not shown).

GCC immunostaining resulted in two cellular localizations, membranous-apical and cytoplasmic; both localizations were evaluated and scored by two independent pathologists who were blinded to the tumor tissue specimens. A specimen was considered positive if a minimum of 10% of tumor cells expressed GCC at any staining intensity.

### Statistical analysis

The correlation of GCC expression between primary tumor and matched liver metastasis was investigated using scatter plots of both H-scores and percentage GCC positivity for both membranous-apical and cytoplasmic expression. All data were presented with descriptive statistics. No formal hypothesis was tested and, therefore, sample size was not statistically determined. The GCC-positive and GCC-negative predictive values (PPV and NPV, respectively) were calculated based on data from the 85 paired primary and metastatic colorectal carcinoma specimens.

## Results

### GCC expression in GI malignancies

**Fig 1:**
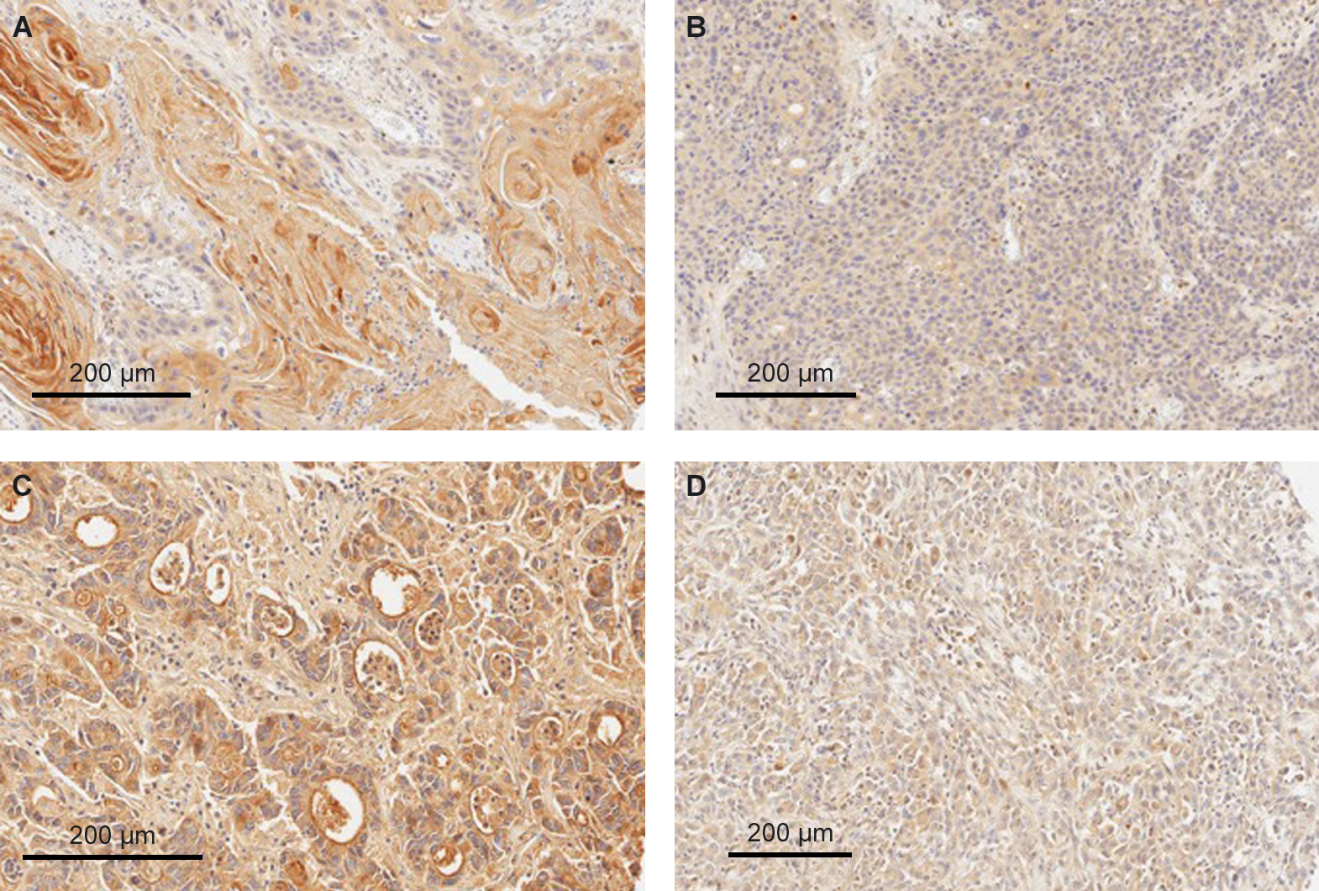
Representative GCC protein expression in GI tumors. (A and B) high and low cytoplasmic expression of GCC in two cases of esophageal squamous cell carcinoma, respectively; (C) strong membranous-apical staining of a pancreatic ductal adenocarcinoma; and (D) weak cytoplasmic staining of a pancreatic adenocarcinoma. All sections are magnified by a factor of 20x. GCC, guanylyl cyclase-C; GI, gastrointestinal.

**Fig 2:**
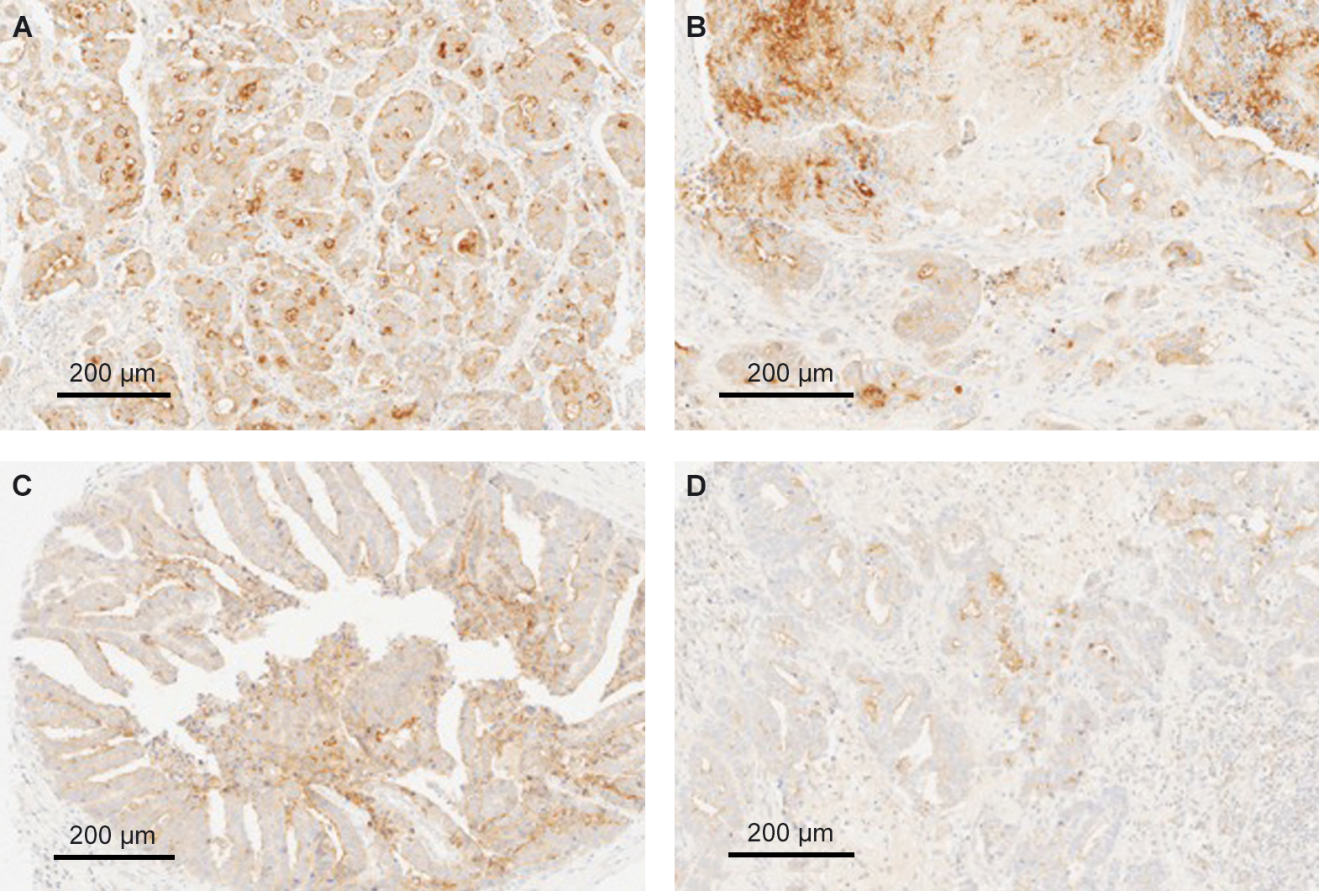
Representative GCC expression in matched primary and metastatic gastric adenocarcinomas. (A and C) two primary gastric intestinal-type adenocarcinomas showing strong membranous-apical staining combined with weak cytoplasmic GCC expression, respectively; (B and D) matching lymph-node metastases from two primary cases showing consistent GCC expression with both membranous-apical and cytoplasmic localization. All sections are magnified by a factor of 20x. GCC, guanylyl cyclase-C.

**Fig 3:**
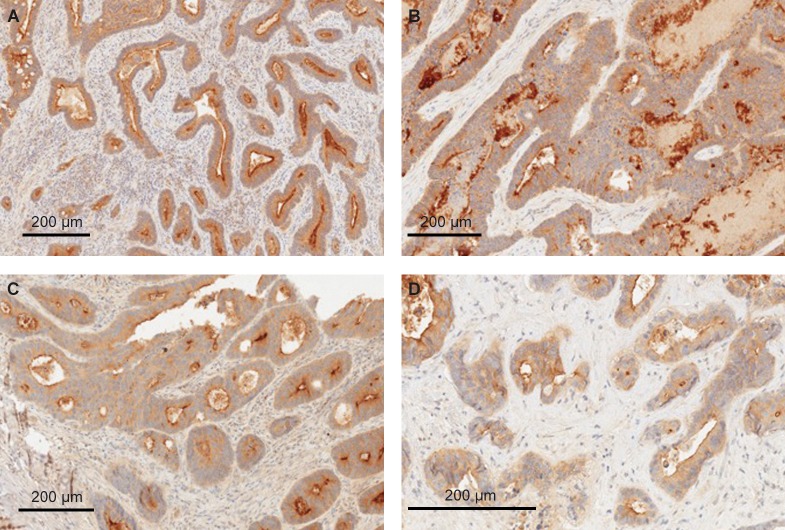
Representative GCC expression in primary CRC and matched liver metastases. (A and C) Strong apical-membrane GCC staining is seen in primary colorectal adenocarcinomas; (B and D) the observed membrane-apical staining is also consistent with observations in the matched liver metastasis. All sections are magnified by a factor of 20x. CRC, colorectal cancers; GCC, guanylyl cyclase-C.

We investigated the GCC protein expression in tumor specimens obtained from a total of 627 individuals with various GI malignancies consisting of both CRC and non-CRC tumors, including esophageal (n = 130), gastric (n = 276; both 180 primary and 96 metastatic), pancreatic (n = 136), and colorectal (n = 85, with matching primary lesions and liver metastasis) tumors. GCC protein expression data and distribution for esophageal, gastric, and pancreatic cancers are shown in Table 1. The frequency of GCC positivity ranged from 59% to 68% in non-CRC tumors. In contrast, CRC specimens showed GCC-positive expression in 98% of primary tumors and 81% of metastatic lesions (Table 2). Representative GCC staining patterns are shown in Figs 1–3.

**Table 1:** GCC status in esophageal, gastric, and pancreatic cancers.

GCC status		Cancer types					
		Esophageal cancers	Gastric cancers (n = 276)				Pancreatic cancers
			Primary			Metastatic	
		Squamous cell (n = 130)	Adenocarcinoma (n = 156)	Signet ring (n = 11)	Undifferentiated (n = 13)	Adenocarcinoma (n = 96)	Adenocarcinoma (n = 136)
GCC negative, n (%)		53 (41)	38 (24)	1 (9)	4 (31)	45 (47)	49 (36)
GCC positive	Total, n (%)	77 (59)	118 (76)	10 (91)	9 (69)	51 (53)	87 (64)
	Cytoplasmic, n (%)	77 (59)	47 (30)	9 (82)	9 (69)	15 (16)	35 (26)
	Membranous-apical, n (%)	0 (0)	13 (8)	0 (0)	0 (0)	14 (15)	12 (9)
	Cytoplasmic + membranous-apical, n (%)	0 (0)	58 (37)	1 (9)	0 (0)	22 (23)	40 (29)

The table reports the numbers of cases with respective GCC status. Percentages are shown in parentheses. The pattern of expression is defined by the numbers of cases with distinct areas in tumor cells that show GCC-positive staining in membranous-apical, cytoplasmic, or both areas.

GCC, guanylyl cyclase-C.

**Table 2:** GCC status in primary CRC and matched liver metastases.

GCC status, n (%)	Primary CRC (n = 85)	Matched liver metastasis (n = 85)
GCC-positive cases, membranous-apical only	0	5 (6)
GCC-positive cases, cytoplasmic only	1 (1)	8 (9)
GCC-positive cases, both membranous-apical and cytoplasmic	83 (98)	69 (81)
GCC-negative cases	1 (1)	3 (4)

CRC, colorectal cancer; GCC, guanylyl cyclase-C.

#### Esophageal cancers

A total of 130 specimens of squamous esophageal cancers were evaluated. We found that 77 (59%) squamous tumors had GCC-positive expression (Table 1). All 77 GCC-positive tumors showed a cytoplasmic staining pattern (Fig 1A and 1B). Membranous-apical staining was absent in all GCC-positive esophageal tumors evaluated.

#### Gastric cancers

A total of 180 primary gastric tumors were examined, which included 156 adenocarcinomas, 11 Signet ring, and 13 undifferentiated tumors. An additional 96 specimens (n = 96 patients) from metastatic lesions (mostly lymph-node metastases) were also tested for GCC expression. Overall, 188 out of 276 (68%) tumors showed GCC-positive expression (Table 1). Among the 96 metastatic cases evaluated, 51 (53%) samples demonstrated GCC positivity (Fig 2B and 2D). The pattern of GCC localization among the primary and metastatic tumors included 80 (29%) cases with cytoplasmic expression. In contrast, 27 (10%) cases among the primary and metastatic tumors tested showed membranous-apical expression. We observed 81 (29%) cases with both membranous-apical and cytoplasmic patterns of GCC expression. (Fig 2A and 2C).

#### Pancreatic cancers

A total of 136 pancreatic adenocarcinomas were examined (Table 1). Overall, we observed 87 (64%) cases that showed GCC-positive expression. Among GCC-positive cases, cytoplasmic expression was detected in 35 (26%) samples, while membranous-apical expression was seen in 12 (9%) samples (Table 1 and Fig 1C and 1D). GCC expression in both membranous-apical and cytoplasmic regions of tumor cells have been detected in 40 (29%) positive cases.

### CRC primary and matched metastatic tumors

GCC expression has been found consistently in both primary and matched metastatic tumors. We observed 98% of primary CRC tumors with both membranous-apical and cytoplasmic patterns of expression (Fig 3A and 3C and Table 2). In contrast, we have identified one case with GCC cytoplasmic expression only and one case that was GCC negative. Similarly, 69 of the 85 (81%) matched metastatic tumor specimens showed staining of GCC in both the membranous-apical and cytoplasmic regions of tumor cells (Fig 3B and 3D). Among a total number of 85 matched metastatic specimens, 5 (6%) demonstrated membranous-apical expression alone, 8 (9%) showed cytoplasmic GCC staining, and 3 (4%) were negative for GCC expression. The pattern of expression in both primary and metastatic tumors tended to be predominantly in both the membranous-apical and cytoplasmic areas of tumor cells (Table 2).

In 41 (48%) cases, the GCC expression was higher in primary than in matched metastatic cancers (Table 3). Comparably, in the primary tumors, 41 out of 85 cases (48%) also showed higher membranous-apical expression than the matched metastatic cancers. In contrast, some matched tumors have shown a lower GCC expression in the primary tumors when compared with the metastatic lesions. Namely, 25 (29%) cases had GCC membranous-apical expression and 34 (40%) had GCC cytoplasmic expression, respectively, that were lower in the primary tumors compared with their matched metastatic tumors (Table 3). Equivalent staining in matched pairs of primary and metastatic tumors was seen in 19 (22%) cases of membranous-apical expression and 10 (12%) cases of cytoplasmic expression.

**Table 3:** Assessment of the relative differences in GCC expression in primary CRC and matching liver metastasis tumor specimens in the distinct areas of tumor cells (membranous-apical and cytoplasmic).

GCC status, n (%)	Membranous-apical GCC-positive status (n = 85)	Cytoplasmic GCC-positive status (n = 85)
Equivalent GCC expression in primary versus metastatic CRC	19 (22)	10 (12)
Higher GCC expression in primary versus metastatic CRC	41 (48)	41 (48)
Lower GCC expression in primary versus metastatic CRC	25 (29)	34 (40)

CRC, colorectal cancer; GCC, guanylyl cyclase-C.

The consistency of GCC expression in primary versus metastatic cancers was further analyzed by assessing both the PPV and NPV of GCC status in primary tumors. Both cytoplasmic and membranous-apical expressions have been analyzed in predicting the GCC status in their matched metastasis. Based on cytoplasmic GCC expression, the PPV and the NPV were 91% and 100%, respectively. Based on the membranous-apical expression, the PPV and NPV were 88% and 73%, respectively. Overall, these data demonstrated that, in the majority of cancer patients, GCC expression in the primary tumor was retained throughout metastatic growth.

## Discussion

In this study, we have evaluated different gastrointestinal cancers for protein expression of GCC. Testing of these tumors by IHC, showed expression of GCC in both the membranous-apical and cytoplasmic region of tumor cells. Our results show positive GCC protein expression in various GI tumors, which is consistent with prior studies [7]. We found GCC protein expression in primary tumors of the esophagus (59%), stomach (68%), CRC (98%), and pancreas (64%).

Squamous cell carcinoma (SCC) and adenocarcinomas are the two main subtypes of cancer of the esophagus, and the squamous subtype primarily occurs in China and the Middle East [16]. To date, most studies evaluating GCC mRNA and protein expressions in esophageal tumors have tested the adenocarcinoma subtype [17, 18]. Our study reports for the first time that SCC tumors express GCC. Recent studies have suggested that esophageal adenocarcinoma and SCC are derived through different processes of tumor evolution and transformation [19]. Namely, adenocarcinomas of the gastroesophageal junction arise from intestinal trans-differentiation promoted by bile acids, with a phenotype incorporating a lineage-specific program of gene expression including GCC [20]. They have more similarities with gastric adenocarcinomas with chromosomal instability rather than esophageal SCC [21]. In contrast, esophageal SCC have been shown to have a different genomic landscape and arise from different precursor lesions through multiple genomic alterations [19]. Our observations that GCC is expressed on the cytoplasmic region of esophageal SCCs, whereas adenocarcinomas show cytoplasmic and membranous-apical expression may reflect different processes of tumorigenesis.

The use of a sensitive and specific IHC assay for GCC allowed for the detection of low levels of membrane-apical expression as well as the detection of cytoplasmic GCC expression. We observed an overall trend of a higher frequency of GCC protein expression in the cytoplasmic region when compared with the membrane-apical region of tumor cells. Further research is needed to investigate biologic implications of localization of GCC and possible clinical consequences. Prior studies have suggested receptor internalization and cycling of receptors between the cytoplasm and cell membrane [22], which could explain the detection of GCC protein in various tumors in both compartments of tumor cells. Our results are in agreement with previous findings, which have shown GCC expression in both primary and metastatic CRC, although possible involvement of GCC on tumor formation needs to be better understood [7, 15, 23].

This study provides further evidence that GCC status of primary cancers correlated with the protein expression of GCC in the matched lymph node or liver metastases in both gastric and CRC tumors respectively. Compared with other primary tumor types which we examined, CRC showed the highest GCC protein expression. Additionally, the intensity of GCC staining is highest in CRC (data not shown). In agreement with prior hypotheses, our data from a series of 85 matched CRC and liver metastasis cases confirm that the GCC-positive status at the protein level remains consistent throughout the process of tumor progression including primary and respective metastases [15]. There were some discrepancies between relative GCC expression in the cytoplasm and the membrane-apical region in the primary lesion and metastases. We show that higher expression of GCC in metastatic versus primary tumors is more common in tumors that are positive for GCC expression in the cytoplasm (40%) compared with membranous-apical-positive tumors (29%). One could hypothesize that in the course of CRC tumor progression, increased internalization of GCC is associated with a loss of activating ligand [24]. Further investigation may lead to a better understanding of the possible impact GCC may have on biologic features and aggressiveness of various GI tumors, which may guide new efforts to develop novel therapeutic modalities based on targeting GCC [13, 25].

In conclusion, this study investigated the protein expression of GCC in a spectrum of GI malignancies. These results identified CRC as the epithelial GI malignancy where GCC is most frequently expressed, when compared with esophageal, gastric, and pancreatic cancers the expression of GCC has been shown for the first time in SCC of the esophagus. Moreover, our findings of GCC protein expression in primary CRC tumors and respective metastases (matched synchronous liver metastases) support a hypothesis that GCC protein expression is maintained throughout various stages of cancer progression.
